# Anxiety, Health Self-Perception, and Worry About the Resurgence of COVID-19 Predict Fear Reactions Among Genders in the Cuban Population

**DOI:** 10.3389/fgwh.2021.634088

**Published:** 2021-03-24

**Authors:** Yunier Broche-Pérez, Zoylen Fernández-Fleites, Evelyn Fernández-Castillo, Elizabeth Jiménez-Puig, Annia Esther Vizcaíno-Escobar, Dunia M. Ferrer-Lozano, Lesnay Martínez-Rodríguez, Reinier Martín-González

**Affiliations:** ^1^Psychology Department, Universidad Central “Marta Abreu” de Las Villas, Santa Clara, Cuba; ^2^CognitiON (Cuban Iniciative on Cognitive Health), Santa Clara, Cuba

**Keywords:** gender, fear of COVID-19, resurgence, anxiety, health self-perception, worry

## Abstract

The resurgence of COVID-19 could deepen the psychological impacts of the pandemic which poses new challenges for mental health professionals. Among the actions that should be prioritized is the monitoring of the groups that have shown greater psychological vulnerability during the first stage of the pandemic. The first aim of our study is to explore the fear reactions to COVID-19 between genders during the second wave of the outbreak in Cuba. Second, establish possible predictors of fear of COVID-19 in relation to gender. Specifically, we will evaluate how anxiety related to COVID-19, health self-perception, and worry about the resurgence of COVID-19 predict fear reactions among women and men in the Cuban population. A cross-sectional online study was designed. The research was conducted between August 16 and October 18, 2020. A total of 373 people completed the online survey. A socio-demographic questionnaire, the Fear of COVID-19 Scale and the Coronavirus Anxiety Scale were used. An independent-samples *t*-test was conducted to compare the fear, worry, anxiety and self-perceived health scores, between genders. The relationship between those variables and fear of COVID-19, was investigated using Pearson correlation coefficient. Finally, multiple linear regression was used to evaluate the possible associations (predictors) related to fear of COVID-19. In our study, women, compared to men, presented greater fear reactions, greater concern about resurgence of COVID-19 and poorer self-perceived health. Anxiety reactions in our sample showed no differences between genders. In women, anxiety of COVID-19, worry about resurgence of COVID-19, and self-perceived health are associated with fear reactions to COVID-19. In the case of men, the self-perceived health showed no associations with fear reactions. Our results confirm the results of several related investigations during the first wave of the pandemic where women have shown greater psychological vulnerability compared to men. However, we cannot rule out that the real impact of the pandemic on mental health in men is much greater than that described by the studies conducted to date. Additional studies are needed on the psychological impact of COVID-19 on men.

## Introduction

Since December 2019 the world has faced a common enemy: the new coronavirus disease 2019 (COVID-19). This new disease (caused by the virus designated as SARS-CoV-2), has constituted an extraordinary challenge for global public health (1).

COVID-19 is also characterized by its fast transmission and high mortality (2). To date (11/12/20), 52 million people have fallen ill with COVID-19, with 1.29 million people dying from the disease (3). The presence of non-communicable chronic diseases (hypertension, diabetes, obesity, chronic renal impairment, etc.), age over 60 years, and the existence of respiratory diseases have been associated with higher mortality rates (4, 5).

In the absence of a definitive cure, measures to contain the spread of the disease include restriction of movement, physical distancing, the establishment of quarantines, the closure of public places (bars, schools, gyms, airports, etc.), and the use of face masks (6, 7).

The measures implemented to contain the pandemic have reduced the growth in the number of cases, preventing the collapse of medical services and saving thousands of lives (8). However, these measures have had a considerable impact on the mental health of the population (9, 10).

In this sense, several studies have been carried out to explore the impact of the pandemic on people's mental health. Among the most frequent mental health problems related to COVID-19 outbreak reported to date are anxiety, insomnia, stress, post-traumatic stress symptoms (PTSS) depression, anger, and fear (10–17).

Currently, the resurgence of the disease in several countries around the world indicates the beginning of the second wave of COVID-19 (18). According to some authors, the second wave of COVID-19 constitutes a significant threat at the social level, exacerbating the impact caused by the first wave in sectors such as the economy and public health (19). Additionally, the resurgence of COVID-19 could deepen the psychological impacts of the pandemic (20), which poses new challenges for mental health professionals around the world. In our opinion, among the actions that should be prioritized at this stage is the monitoring of the groups that have shown greater psychological vulnerability during the first stage of the pandemic.

Women are among the most vulnerable groups in terms of mental health during the current pandemic. The greater vulnerability of women, in comparison to men, in terms of mental health during the current pandemic is related to biological, psychological and sociocultural factors (21). For example, from a biological perspective in situations of acute stress, women show a lower adaptation to hypersecretion of the corticotropin-releasing factor (CRF) (2), making them more vulnerable to developing mental disorders involving hyperarousal (22).

This pattern of response to stress has an expression in the prevalence of mental disorders that are diagnosed more frequently in women. Persistent exposure to stressful situations increases the vulnerability to develop posttraumatic stress disorder, panic disorder, and major depression, disorders that are diagnosed more frequently in women (23). These differences between women and men in terms of the psychopathological profile have been previously reported in non-pandemic times. For example, in response to situations of chronic stress, women generally develop phobia, depression, anxiety, and panic disorders more frequently than men (24).

However, the risk that the current pandemic poses to women's mental health cannot be understood solely in biological terms. In addition, there are socio-cultural factors that also create a gap between genders. For example, around 70% of healthcare and social services workforce are women, increasing the risk of infection by the virus (25) unlike men who are generally exposed to the virus in a non-health care setting (26). Additionally, in countries like Singapore and Germany more women than men have lost their jobs during the current pandemic and also a greater number of women have seen their formal work hours reduced and their domestic work hours increased (27).

In the long term, being unemployed has a negative impact on mental health, increased relative-risk of death by suicide, compared with being employed (28). On the other hand, during the current pandemic caregiver responsibilities have increased, especially after the closure of childcare centers (21), potentially increasing stress and negatively impacting psychological well-being, especially in women (29). This scenario places women in a position of increased vulnerability during the current pandemic, having a negative impact on their mental health.

Several studies to date have found that women, compared to men, have experienced higher levels of psychological discomfort, depression, anxiety, psychological distress, insomnia, adjustment disorder, and fear related to COVID-19 (16, 30–33).

Fear has been one of the most studied emotional reactions during the current outbreak. This emotion is defined as an unpleasant state due to the perception of threat (34) acting as an intervening variable between a set of context-dependent stimuli and suites of behavioral response (35). During the pandemic, fear has shown an adaptive function by stimulating self-care behaviors (36, 37). However, high levels of fear increase distress and anxiety, increasing vulnerability to develop mental illness (34).

In this sense, it has been proven that women are more susceptible to developing disorders related to fear responses compared to men (38). A tentative etiological explanation for this phenomenon has been the existence of a longer extinction duration of fear generalization in women (39). These results have also been supported by neuroimaging studies. In the case of women, during the fear conditioning process, greater BOLD-signal changes have been observed the right amygdala, right rostral anterior cingulate (rACC) and dorsal anterior cingulate cortex (dACC) (40). This characteristic would contribute to a greater risk to the mental health of women compared to men when they are exposed to stressful situations that evoke fear responses.

In the literature, different predictors of fear reactions have been described in the general population. For example, it has been shown that anxiety, intolerance of uncertainty, perceived infectability, worry, media exposure, and depression has been associated with fear of COVID-19 in the general population (41–46).

## The Current Study

The first case of COVID-19 in Cuba was reported on March 11, 2020. To date (26/01/2021), more than 22 600 cases positive of COVID-19 have been reported in Cuba and 200 people have died from this disease (47).

During the first 4 months of the fight against the pandemic, the measures of the Cuban government and the Ministry of Public Health included the mandatory use of the face mask, the closure of international borders, the closure of schools, and the establishment of quarantines in places with significant outbreaks of the disease (Cuba's COVID-19 strategy, 2020). These measures made it possible to significantly reduce the number of positive cases throughout the country.

However, since August 2020, the country has experienced a sustained increase in the number of positive cases for the disease. In the first 8 days of August alone, 255 cases were confirmed, representing 90% of the confirmed cases in July (Ministry of Public Health, 2020). Although the use of face masks and social distancing remain mandatory, the authorities will no longer isolate those who have been in contact with suspected cases, school activities are restarted, airports are open and economic activity in the country is reactivated.

This scenario constitutes a major challenge for mental health, as people must return to their daily activities even when there is no cure for the disease, potentially increasing fear and anxiety reactions, especially in the most vulnerable groups.

In Cuba, a study conducted by Broche-Pérez et al. (33) during the first wave of the disease found a greater fear response related to COVID-19 in women. However, the authors did not explore predictors of fear reactions in the sample, making it difficult to design interventions that reduce the negative impact of the outbreak on the mental health of women and men. The design of interventions is very important in the Cuban context considering that the country is already facing the resurgence of COVID-19.

In this study we will focus specifically on how anxiety related to COVID-19, health self-perception and worry about the resurgence of the outbreak are related to fear reactions to COVID-19 in the Cuban population. Although the terms fear, anxiety and worry are sometimes used synonymously, they are separate constructs. Both fear and anxiety are the result of adaptive defensive behavior that aims to escape a threat or motivational conflict (48). However, according to Öhman (49) fear reactions denotes dread of impeding, disaster and an intense urge to defend oneself, and anxiety has been described as an ineffable and unpleasant foreboding. On the other hand, worry and anxiety should also be considered as separate constructs (50). While worry is related to problem-focused and adaptive coping strategies, anxiety is associated with negative affect (51).

The first objective of our study is to explore the fear reactions to COVID-19 between genders during the second wave of the outbreak in Cuba. Second, to establish possible predictors of fear of COVID-19 (anxiety related to COVID-19, health self-perception, and worry about the resurgence of COVID-19) among women and men in the Cuban population.

## Materials and Methods

### Study Design and Participants

A cross-sectional online study was designed. To disseminate the survey, the Google Forms^®^ platform was used. The survey was released through social media (Facebook, WhatsApp, and Telegram). An announcement of the study was also published on the website of the Wellbeing Center of the Universidad Central “Marta Abreu” de Las Villas. The research was conducted between August 16 and October 18, 2020. All Cuban citizens over 18 years were eligible. A total of 373 people completed the online survey. For this sample size, a power analysis was running (*post hoc*) using the G*Power software (version 3.1.9.2) (52). Considering the statistical test (multiple linear regression), and the number of predictors (three predictor) the sample showed a power of.99.

### Instruments

#### Background Information

The demographic variables explored included the age, gender and education. To evaluate health self-perception, we use the *ad hoc* question “how do you consider your health is?” [from 1 (“very poor”) to 5 (“excellent”)]. To explore worry about the resurgence of COVID-19 we used the *ad hoc* question “how concerned are you about the resurgence of COVID-19?” [from 1 (“not at all”) to 5 (“very concern”)].

#### The Fear of COVID-19 Scale

The Fear of COVID-19 Scale (FCV-19S) (41) is made up of seven items with a five-item Likert-point response from 1 (“strongly disagree”) to 5 (“strongly agree”). The score range of the FCV-19S is 7 to 35. Higher scores indicate greater fear of COVID-19. In this study, the Cuban version of the scale was used (53). For the Cuban population the Cronbach alpha coefficient was 0.80.

#### The Coronavirus Anxiety Scale

The Coronavirus Anxiety Scale (CAS) (54) was developed to assess the anxiety reactions related to COVID-19 pandemic. The CAS consists of 5 items with a Likert-point response from 0 (“not at all”) to 4 (“nearly every day over the last 2 weeks”). The original version of CAS has excellent internal consistency, with a Cronbach alpha coefficient reported of 0.93. For the Cuban population the Cronbach alpha coefficient was 0.88 (33).

### Procedure

The study protocol was approved by the ethics committee of the Department of Psychology of the Universidad Central “Marta Abreu” de Las Villas. All procedures performed in this study were in accordance with the ethical standards of the 1964 Helsinki Declaration. Informed consent was obtained from all participants included in the study.

### Data Analysis

The data were processed using SPSS/Windows (version 21). Descriptive statistics was used to explore participants' characteristics. An independent-samples *t*-test was conducted to compare the fear of COVID-19 scores, worry about resurgence of COVID-19, anxiety related to COVID-19 and self-perceived health between genders. Results with *p* < 0.05 were regarded as significant. The Cohen's d were calculated to estimate effect sizes in all comparisons. Values above 0.2, 0.5, and 0.8 were considered as small, medium, and large effect size, respectively (55). The relationship between fear of COVID-19, anxiety related to COVID-19, worry about resurgence of COVID-19 and self-perceived health was investigated using Pearson product-moment correlation coefficient. Finally, multiple linear regression was used to evaluate the effects of worry about resurgence of COVID-19, anxiety related to COVID-19 and self-perceived health over fear of COVID-19 scores.

## Results

### Characteristics of Participants

Table 1 shows the characteristics of the participants. The mean age of participants (*n* = 373) was 32.1 years, with a range between 18 years to 81 years old. In the study women predominate, representing 63.9% of the participants. Most of the participants (76.7%) finished college. In the sample, 279 participants (74.8%) rated their health as “very good” or “excellent.” On the other hand, 291 participants (78%) expressed feeling “concerned” or “very concerned” with the second wave of COVID-19.

**Table 1:** Characteristics of survey participants (*N* = 373).

**Characteristics**	**Fr (%)**
Age [M(SD)]	32.1 (12.9)
Gender	
Female	238(63.8)
Male	135 (36.2)
Education status	
Primary school	6 (1.6)
Secondary school	3 (0.8)
Higher secondary level	78 (20.9)
Tertiary education	286 (76.7)
Self-reported health status	
Very poor	1 (0.3)
Poor	7 (1.9)
Average	86 (23.1)
Very good	214 (57.4)
Excellent	65 (17.4)
Worry about outbreak	
Not at all	8 (2.1)
Not too much	50 (13.4)
I can say	24 (6.4)
Concern	214 (57.4)
Very concern	77 (20.6)

*M, mean; SD, standard deviation; fr, frequency*.

### Comparisons Between Genders on the Fear of Coronavirus-19 Scale and Related Variables

The results of the comparison of variables between groups are shown in Table 2. There are significant differences in fear of COVID-19, worry about resurgence of COVID-19 and self-perceived health between genders. The female participants showed higher levels of fear of COVID-19 and worry about resurgence. Women also perceived their health status as poorer compared to men. No differences between female and male participants were found in anxiety related to COVID-19.

**Table 2:** Comparisons between genders on the Fear of Coronavirus-19 Scale and related variables.

	**Female (*n* = 238)**	**Male (*n* = 135)**			
**Variables**	**M (SD)**	**M (SD)**	***t***	***p***	***d***
Fear of COVID-19	19.02 (5.93)	17.34 (6.29)	2.566	0.01	0.27
Anxiety related to COVID-19	8 (3.51)	7.24 (3.89)	1.905	0.058	0.20
Worry about resurgence of COVID-19	3.97 (0.85)	3.52 (1.12)	4.413	<0.001	0.47
Self-perceived health	3.81 (0.67)	4.05 (0.72)	3.218	0.001	0.35

*M, mean; SD, standard deviation*.

### Correlations Between Variables

The relationship between fear of COVID-19, anxiety related to COVID-19, worry about resurgence of COVID-19 and self-perceived health was investigated using Pearson product-moment correlation coefficient (Table 3). Preliminary analyses were performed to ensure no violation of the assumptions of normality, linearity and homoscedasticity. There was a strong, positive correlation between the fear of COVID-19, anxiety related to COVID-19 and worry about resurgence of COVID-19, with high levels of fear of COVID-19 associated with high levels of anxiety and worry about resurgence of COVID-19. It was also found a negative correlation between the fear of COVID-19 and self-perceived health with high levels of fear associated with lower levels of self-perceived health.

**Table 3:** Correlations between gender on the Fear of Coronavirus-19 Scale and related variables.

**Gender**	**1**	**2**	**3**	**4**	
Male	1.Fear of COVID-19	-			
	2.Anxiety related to COVID-19	684^**^	-		
	3.Worry about resurgence of COVID-19	365^**^	0.110	-	
	4.Self-perceived health	-428^**^	-459^**^	-261^**^	-
Female	1.Fear of COVID-19	-			
	2.Anxiety related to COVID-19	632^**^	-		
	3.Worry about resurgence of COVID-19	482^**^	349^**^	-	
	4.Self-perceived health	-141^*^	−0.033	−0.038	-

** *Correlation is significant at the 0.01 level (2-tailed);*

* *Correlation is significant at the 0.05 level (2-tailed)*.

There is a statistically significant difference in the strength of the correlation between fear of COVID-19 and self-perceived health for males and females (*z* = −2.90; *p* = 0.003). The correlation between fear of COVID-19 and anxiety (*z* = 0.84; *p* = 0.40) and between fear of COVID-19 and worry about resurgence of COVID-19 (*z* = −1.32; *p* = 0.18) did not show significant differences in the strength of the correlations between the groups.

### Regression Analysis

A multiple regression analysis was run to predict fear of COVID-19 levels between genders from anxiety related to COVID-19, worry about resurgence of COVID-19 and self-perceived health. The Table 4 shows that the independent variables statistically significantly predict the dependent variable (for both groups) (male: F_(3, 131)_ = 54.766, *p* < 0.0001, *R*^2^ = 0.55; female: F_(3, 234)_ = 74.928, *p* < 0.0001, *R*^2^= 0.49). In the case of women, the three variables included in the analysis made it possible to predict fear reactions in this group (*p* < 0.05). In the case of men, the variables anxiety related to COVID-19 and worry about resurgence of COVID-19 showed associations with fear reactions in this group (p <0.001). However, self-perceived health was not a predictor of fear reactions to COVID-19 in the case of men (*p* = 0.30).

**Table 4:** Predictors of Fear of COVID-19.

**Gender**		**B**	**SE**	**β**	***t***	**Sig**.
Male	(Constant)	7.086	3.269		2.168	0.032
	Anxiety related to COVID-19	1.003	0.106	0.621	9.487	0.000
	Worry about resurgence of COVID-19	1.551	0.336	0.278	4.610	0.000
	Self-perceived health	−0.607	0.583	−0.070	−1.040	0.300
Female	(Constant)	7.551	2.097		3.602	0.000
	Anxiety related to COVID-19	0.888	0.084	0.526	10.555	0.000
	Worry about resurgence of COVID-19	2.047	0.348	0.294	5.890	0.000
	Self-perceived health	−0.988	0.410	−0.113	−2.410	0.017

*Dependent Variable: Fear total score; SE, standard error*.

## Discussion

The first objective of our study was to explore the fear reactions to COVID-19 between genders during the second wave of the outbreak in Cuba. Second, to establish possible predictors of fear of COVID-19 in relation to gender. In our study, women, compared to men, presented greater fear reactions, greater concern about resurgence of COVID-19 and poorer self-perceived health. The anxiety reactions between men and women did not show significant differences.

Additionally, we verified that *anxiety related to COVID-19, concern about the resurgence of COVID-19* and *self-perceived health* showed associations with the fear response in the case of women. In the case of men, the *self-perceived health* variable does not predict fear reactions.

Several studies conducted during the pandemic have confirmed a greater psychological vulnerability in women compared to men. For example, research has confirmed in women a greater experience of fear, post-traumatic stress symptoms (PTSS), adjustment disorder anxiety, depression and anxiety (9, 16, 32, 33, 56–59).

In the specific case of fear, our study supports the results reported by Broche-Pérez et al. (33) during the first wave of the disease in Cuba. In our study, women also showed a greater fear reaction compared to men. The mean reported by Broche-Pérez et al. (33) in the first study was 17.9 (SD ± 8.05) for men and 21.9 (SD ± 6.9) for women. In our study, the mean for men was 17.34 (SD ± 6.29) and 19.02 (SD ± 5.93) for women. We also found that women showed poorer self-perceived health compared to men, and that self-perceived health values have an inverse relationship with fear reactions (better self-perceived health is related to less fear reaction). This result is consistent with studies showing that, around the world, women tend to rate their self-perceived health as weaker compared to men (60). This result is important because it has been proven that poorer self-perceived health is related to poorer mental health. For example, more self-perceived health has been associated with a greater experience of depression, anxiety and psychological distress in various populations (61–65).

During the pandemic, it has been reported that the lowest levels of self-perceived health reported by women have been related to residing in places with a high prevalence of the disease (66), however, more studies are needed to explore the relationship between self-perceived health and mental health outcomes during the current pandemic. The gap between women and men in relation to self-perception of health has been attributed in part to social factors (gender inequality index, education or employment) rather than to behavioral factors (67).

On the other hand, worry about resurgence was also higher in women compared to men. In the past, worry have been related to depressive rumination (31) generalized anxiety (6), panic disorder, and obsessive-compulsive disorder (30). During the pandemic, elevated levels of worry have been related to higher levels of anxiety, stress, intrusive thoughts, avoidance and fear of mental health (1, 68). However, greater worry in women can also have a positive effect by stimulating safety behaviors, such as the use of personal protective equipment (68). In the Cuban context, the concerns related to COVID-19 during the second wave could be closely related to the return to daily life, the opening of schools, airports, and the increase in the number of infections compared to the first wave.

These results show a greater vulnerability in women compared to men. This results may have several tentative explanations, both from a biological, and sociocultural perspective. For example, there is evidence on the differences between women and men in relation to reactivity to stress, which is related to the prevalence and presentation of many psychiatric disorders (2, 22, 69). For example, women more frequently develop mental disorders in response to situations involving hyperarousal (22), closely related to fear responses. This vulnerability in women could be related, among other factors, to a lower adaptation of women to hypersecretion of the orticotropin-releasing factor (CRF) (2).

On the other hand, there are also sociocultural factors that increase the vulnerability of women during the current pandemic. For example, gender roles (70, 71), long-existing inequalities, and social disparities (72) are among the factors that exacerbate the impact on mental health in women during the current pandemic.

However, in our opinion the fact that most of the studies conducted during the pandemic report a greater psychological impact of the outbreak on women does not mean that men are exempt from risk. In fact, we cannot rule out that the real impact of the pandemic on mental health in men is much greater than that described by the studies conducted to date. This is especially important in countries where the social construction of gender is structured around a “hegemonic masculinity.” For example, in Latin America the term “machismo” refers a set of attitudes and identities associated with the concept of masculinity (73). This implies that men to be “really men” must suppress their emotions, they must not worry about their health, they must show great inner strength and they must have self-control (74). This social construction of masculinity has a negative impact on the general health and mental health of men (73, 75, 76), even when the results of most research during the pandemic place greater emphasis on the vulnerability of women.

The results obtained must be considered when designing interventions to reduce the impact of the current pandemic on the mental health of Cuban women. Interventions should be implemented as soon as possible, because evidence suggests that delays in receiving psychological treatment result in higher rates of baseline negative psychological symptoms (77). To date, in Cuba, psychological intervention actions have been carried out using telepesychology, both in its synchronous mode (telephone counseling, and support groups through WhatsApp) and asynchronous (design of mobile applications and self-help bulletins) (78, 79). For example, from the Community Mental Health Centers and the Women and Family Orientation Houses, support groups could be implemented where women receive training in stress and anxiety management.

However, to date we are not aware of other studies carried out in Cuba during the current pandemic in which other types of interventions such as cognitive behavioral therapy (CBT) were implemented. The use of brief cognitive behavioral interventions is of great importance, above all because of its proven effectiveness in disorders related to anxiety and fear responses (80). There are international experiences that have implemented CBT during the current outbreak, demonstrated effectiveness in reducing psychological distress in vulnerable populations, including women (77).

Our study presents some limitations that must be discussed. First, the study sample is relatively small. The size of our sample is fundamentally due to the difficulties in internet connectivity that still exist in the country and to the prices of the service. This causes that many potential participants who receive the survey do not complete it. It is possible that participants with a medium or high socioeconomic status predominate in our sample, however this variable was not explored. In this sense, in future studies it would be convenient to explore the socioeconomic status of the participants included in the study. In future studies, other variables related to the family should also be explored in greater depth, such as the quality of the health of close relatives (children, grandparents, etc.). This variable would allow a better understanding of fear reactions in the Cuban population. On the other hand, the presence of psychiatric antecedents in the participants must also be studied in depth, which may explain the variability in fear reactions among Cubans. It would also be interesting to stratify the sample by educational levels, considering that in our study most of the participants have tertiary education, which has been related to better general health (81). Despite these limitations, we consider that our study offers a first approach to the mental health of the Cuban population with an emphasis on gender differences.

## Conclusions

In conclusion, during the second wave of COVD-19 in Cuba, women show greater psychological vulnerability compared to men. The women reported greater experiences of fear, greater concern about the resurgence of COVID-19 and worse self-perceived health. The anxiety reactions between genders did not show significant differences in our sample. Additionally, we found that the variables anxiety, concern for the resurgence and self-perceived health allow predicting the response to fear in the case of women. In the case of men, the self-perceived health variable did not constitute a statistical predictor of the level of fear. It is important to delve into the psychological impact of the pandemic on men, considering that some characteristics related to the social construction of gender could mask the reality of mental health in this gender. Our results will allow the design of interventions in the Cuban context considering that the country is already facing the resurgence of COVID-19.

Interventions must be designed and implemented briefly and must also use evidence-based techniques that are culturally adapted to the Cuban context.

## Data Availability Statement

The datasets presented in this study can be found in online repositories. The names of the repository/repositories and accession number(s) can be found at: http://dx.doi.org/10.17632/srtb8gnrkp.1.

## Ethics Statement

The studies involving human participants were reviewed and approved by Universidad Central Marta Abreu de Las Villas, Department of Psychology. The patients/participants provided their written informed consent to participate in this study.

## Author Contributions

YB-P analyzed the data, and was responsible for preparing the first draft of the article. All authors contributed to the study design. All authors contributed to the article and approved the submitted version.

## Conflict of Interest

The authors declare that the research was conducted in the absence of any commercial or financial relationships that could be construed as a potential conflict of interest.
